# The Relationship Between Situational Motivation and the Effect of Verbal Encouragement on Long Jump Performance: Autonomous vs. Controlled Motivation

**DOI:** 10.3390/sports14050193

**Published:** 2026-05-07

**Authors:** Amir Romdhani, Ahmed Ghorbel, Ghada Regaieg, Vlad Adrian Geantă, Alexandra Reta Iacobini, Alexandru Ioan Băltean, Makram Zghibi, Omar Trabelsi

**Affiliations:** 1 Department of Education and Teaching, Higher Institute of Human Sciences, University of Jendouba, Jendouba 8100, Tunisia; romdhaniamir@isshj.u-jendouba.tn; 2 Research Unit: Physical Activity, Sport, and Health, UR18JS01, National Observatory of Sport, Tunis 1003, Tunisia; ahmed.ghorbel@isseps.usf.tn; 3 The High Institute of Sport and Physical Education, University of Jendouba, Kef 7100, Tunisia; ghada.regaieg93@gmail.com (G.R.); makram.zghibi@issepkef.u-jendouba.tn (M.Z.); 4 Department of Physical Education and Sport, Faculty of Physical Education and Sport, Aurel Vlaicu University of Arad, 310330 Arad, Romania; vlad.geanta@uav.ro; 5 Department of Physical Education and Sport, Faculty of Physical Education and Sport, Spiru Haret University, 030045 Bucharest, Romania; alexandra.iacobini@spiruharet.ro

**Keywords:** verbal encouragement, situational motivation, intrinsic motivation, performance enhancement, athletics, sport psychology

## Abstract

This study examined whether pre-existing situational motivation correlates with the magnitude of performance improvement elicited by verbal encouragement (VE) in long jump. A total of 134 physically active sports science students (21.1 ± 1.4 years) performed a long jump task under two conditions: with and without peer VE. Situational motivation, differentiated into autonomous (Intrinsic Motivation [IM], Identified Regulation [IR]) and controlled (External Regulation [ER], Amotivation [AM]) forms, was assessed immediately prior to trials using the Situational Motivation Scale (SIMS). Performance improvement was calculated as the percentage change (Δ%) between conditions. Paired *t*-tests evaluated the overall effect of VE, while multiple linear regression analysis was used to assess the predictive relationship between situational motivation subscales and performance gains (Δ%), with sex included as a covariate. The results showed that VE significantly enhanced performance across both sexes (*p* < 0.001; d = 1.109–1.331). The regression models indicated that Δ% was positively predicted by autonomous forms of motivation (IM: R^2^ = 0.252; IR: R^2^ = 0.262) and negatively predicted by controlled forms (ER: R^2^ = 0.27; AM: R^2^ = 0.249). Sex was not a significant predictor in any model (*p* > 0.05), indicating that all observed relationships were consistent across both male and female participants. These findings indicate that the performance-enhancing effect of VE in long jump is associated with the initial motivational state of the practitioners, being greater in autonomously motivated individuals and attenuated in those with controlled motivation. Consequently, situational motivation should be assessed before implementing VE in long jump, as its effectiveness is limited in individuals with low autonomous drive and may require preliminary strategies to enhance task engagement.

## 1. Introduction

Athletic performance results from dynamic interaction of physical, technical, and psychological determinants. Among these, motivation plays a pivotal role in enabling athletes to exert effort, maintain focus, and withstand the demands of training and competition [1]. In track and field disciplines, particularly explosive, single-attempt events such as the long jump, the influence of motivation may be especially pronounced due to the performance pressure and technical precision required for optimal execution [2].

Importantly, beyond general motivational disposition, situational motivation at the moment of task engagement may critically influence responsiveness to external interventions [3]. Situational motivation refers to the athlete’s moment-to-moment drive to engage in a specific activity [4]. Unlike trait motivation, which reflects a stable disposition, situational motivation is context-dependent and fluctuates according to task demands, environmental cues, and psychological states [5]. In sport settings, it shapes the intensity of effort, attentional focus, and persistence displayed during performance [3,4].

According to Self-Determination Theory (SDT) [6], situational motivation can be categorized into autonomous and controlled forms [7,8]. Autonomous motivation arises when athletes engage in an activity out of intrinsic interest or personal value, whereas controlled motivation reflects engagement driven by external pressures, obligations, or contingencies. Autonomous forms of motivation are consistently associated with greater engagement, performance quality, and psychological well-being [9], while controlled motivation, although capable of producing short-term effort, is generally less stable and may be linked to anxiety and reduced enjoyment [10].

Verbal encouragement (VE) is widely used to enhance effort and performance in sport contexts. It involves spoken cues or feedback delivered by coaches, peers, or support staff to increase engagement during training or competition [11]. From an SDT perspective, need-supportive communication may facilitate internalization processes and strengthen volitional engagement [1]. Motor learning frameworks further clarify potential mechanisms. The OPTIMAL theory and the constrained action hypothesis suggest that VE may enhance performance by promoting adaptive attentional focus and reducing excessive conscious control, thereby facilitating more automatic and efficient motor execution [12].

Empirical research provides substantial evidence supporting the efficacy of VE across diverse sports and populations. In team sports, VE has been shown to enhance both physiological load and technical performance. For example, Selmi et al. [13] reported that VE during 4v4 small-sided soccer games increased mean and maximal heart rate, as well as technical indicators such as successful passes and interceptions. Similarly, Yilmaz et al. [14] found that VE elevated heart rate responses, ratings of perceived exertion (RPE), enjoyment, and technical actions (e.g., passes and shots) in youth basketball players.

The benefits of VE also extend to individual contexts. During repeated agility training, VE was associated with higher internal load, lactate concentration, and more positive affective responses [15]. In fitness testing environments, Pacholek and Zemková [16] demonstrated improvements in sprint and vertical jump performance when VE was combined with feedback and goal-setting strategies. Romdhani et al. [17] showed that peer-delivered VE elicited greater performance and psychophysiological responses than coach-delivered VE. Endurance performance appears similarly responsive, as Net et al. [18], observed improvements in VO_2_ max estimates, distance covered, and heart rate during the multistage shuttle run test when systematic VE was provided.

Despite this broad support, VE effectiveness is not uniform and appears influenced by contextual and interpersonal factors, including feedback source, content, and delivery style. Mastrich et al. [19] reported that supportive and corrective feedback enhanced intrinsic motivation, mediating competitive performance improvements. Likewise, Romdhani et al. [17] suggested that peer-delivered VE may be more impactful than coach-delivered VE, potentially due to greater social identification. Furthermore, combining VE with attentional focus strategies may amplify its performance effects [12].

However, although VE has been widely examined in relation to physiological and performance outcomes, its relationship with pre-existing situational motivation at the moment of task engagement remains largely unexplored. Most studies infer motivational mechanisms without directly assessing participants’ motivational states to the athletic task being studied. It is therefore possible that VE responsiveness is associated with situational motivation, with performance gains varying according to the practitioner’s pre-existing motivational state relative to the athletic task.

Therefore, the present study investigates the relationship between situational motivation and the magnitude of VE-induced performance enhancement in a long jump task among physically active practitioners. Given that situational motivation fluctuates across tasks and contexts, we hypothesized that VE effectiveness would be predicted by the specific underlying reasons for which practitioners engage in the long jump task. Specifically, higher autonomous motivation was expected to correlate with greater performance gains, whereas higher controlled motivation was expected to be associated with reduced responsiveness to VE.

## 2. Materials and Methods

### 2.1. Participants

A priori power analysis was conducted using G*Power (version 3.1.9.7; Heinrich Heine University Düsseldorf, Düsseldorf, Germany) [20] to determine the sample size required for a multiple linear regression analysis. Based on a fixed model (R^2^ deviation from zero) with three predictors (including the specific SIMS subscale score, sex as a covariate, and the corresponding sex × motivation interaction), a medium effect size (f^2^ = 0.15), an alpha level of 0.05, and a power of 0.95, the analysis indicated that a minimum of 119 participants was necessary.

**Figure 1:**
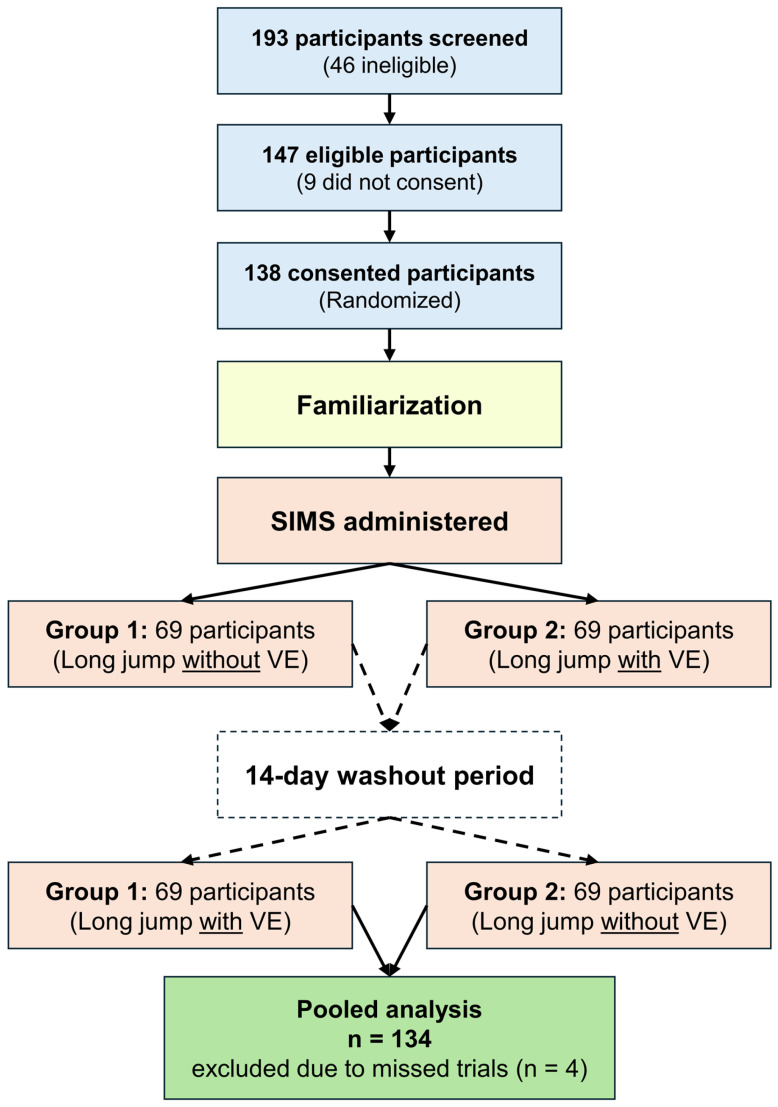
Flowchart of participant enrollment, randomization, and analysis. SIMS, Situational Motivation Scale; VE, verbal encouragement.

Following multiple recruitment visits and advertisements at the High Institute of Sports and Physical Education, Kef, Tunisia, 193 physically active individuals were initially screened (see Figure 1). Of these, 147 met the eligibility criteria, and 138 provided informed consent.

After exclusions during the study (n = 4), the final sample consisted of 134 physically active practitioners (69 males, 65 females; mean age = 21.1 ± 1.4 years). Males averaged 178.5 ± 6.5 cm in height and 74.2 ± 7.8 kg in body mass, whereas females averaged 165.8 ± 6.1 cm and 61.4 ± 6.5 kg.

Eligibility criteria required enrollment in an undergraduate sports science program that included a minimum of 10 h of practical athletic training sessions (e.g., athletics, gymnastics, team sports, combat sports, etc.) at the time of data collection. To ensure homogeneity in task familiarity and training exposure, individuals were excluded if they specialized in long jump or a comparable discipline within their curriculum, or if they engaged in extracurricular sports training (professional club participation or recreational training such as gym membership) within the previous six months or during the study period.

Participants were specifically selected from a population of sports science students who were not specialized in the long jump or involved in high-level extracurricular sports training. This exclusion criterion was implemented to ensure a heterogeneous distribution of situational motivation scores across the sample. By avoiding a cohort of specialized athletes (who typically demonstrate high, uniform levels of autonomous motivation), we were able to analyze the intervention’s effects across a wider variety of motivational profiles, including those with higher levels of external regulation and amotivation.

The study was approved by the Institutional Review Board (IRB) of the High Institute of Sports and Physical Education of Kef, Tunisia (protocol code: SP-0026/2024, approval date: 2 December 2024) and conducted in accordance with the Declaration of Helsinki (2013). Formal authorization was obtained to access the institutional track and field facilities. All participants provided written informed consent prior to participation and were informed of their right to withdraw at any time without penalty. For methodological purposes, the specific athletic task was not disclosed during recruitment; this procedural blinding was explicitly stated in the consent document.

### 2.2. Measures

#### 2.2.1. Effect of Verbal Encouragement on Athletic Performance

The long jump was selected to assess the effects of verbal encouragement (VE) on explosive performance. This task involves a maximal acceleration phase, a take-off from a designated board, and landing in a sandpit. Its short-duration, high-intensity nature allows efficient testing of multiple participants within a session.

**Figure 2:**
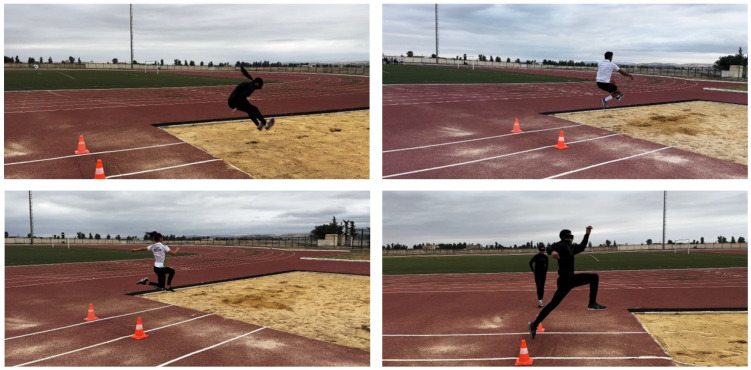
Illustration of the long jump experimental task in the track-and-field environment used for data collection.

Standard field dimensions were used, including a 40 m runway, a 20 cm take-off board, and a landing sandpit approximately 9 m long and 2.75 m wide, in accordance with international track and field guidelines (see Figure 2).

Participants completed a standardized 15 min warm-up consisting of 5 min of jogging, 5 min of dynamic stretching, and 5 min of task-specific drills. Two unrecorded practice trials were performed to ensure readiness.

Each participant then completed three official trials per condition. Additional attempts were permitted only if all initial trials were fouls. A 5–7 min rest interval was provided between trials to ensure adequate recovery.

Jump distance was measured from the nearest mark in the sand to the take-off board, perpendicular to the board, using a calibrated decameter tape. The longest valid jump was retained as the performance outcome for each condition.

#### 2.2.2. Situational Motivation

Situational motivation was assessed immediately prior to testing using the Situational Motivation Scale (SIMS), originally developed by Guay et al. [7]. The SIMS evaluates motivation toward a specific activity at a given moment, based on Self-Determination Theory (SDT) [6].

The scale consists of 16 items, with four items for each of the four key motivational sub-scales: “Intrinsic Motivation” (IM), “Identified Regulation” (IR), “External Regulation” (ER), and “Amotivation” (AM). Participants respond to each item on a 7-point Likert scale ranging from 1 (“Does not correspond at all”) to 7 (“Corresponds exactly”).

The SIMS asks participants a single question: “Why are you currently engaged in this activity”? The participants then respond to this question by rating their agreement with the statements of the four subscales. The statements of “Intrinsic Motivation” assess the extent to which participants are engaging in the task for the pleasure and satisfaction they derive from the activity itself (e.g., “Because this activity is exciting”). In the same line, the statements of “Identified Regulation” measure motivation that stems from a personal value or goal, even if the activity itself is not inherently enjoyable (e.g., “Because I think this activity is beneficial for me”). On the other hand, the statements of “External Regulation” gauge motivation driven by external pressures, such as rewards, punishments, or social approval (e.g., “Because it is something that I have to do”). Finally, the statements of “Amotivation” reflect a lack of motivation, where participants feel a sense of incompetence or a lack of purpose for engaging in the activity (e.g., “I don’t know; I don’t see what this activity brings me”).

To ensure cultural and linguistic appropriateness of the SIMS, a cross-cultural validation process was conducted six months prior to the main study. The scale was first translated from English to Arabic following standard forward–backward translation procedures, with expert review to resolve semantic discrepancies. A pilot test was then carried out on 35 potential participants drawn from the target population to examine clarity and comprehension. Internal consistency of the Arabic version was satisfactory, with Cronbach’s alpha values ranging from 0.78 to 0.87 across subscales. Test–retest reliability, assessed on the same pilot group after a two-week interval, demonstrated good temporal stability (intraclass correlation coefficients = 0.82 – 0.89).

The specific nature of the activity (long jump) was withheld during recruitment to prevent self-selection bias. By avoiding the targeted enrollment of individuals with high task-specific interest, we ensured sufficient variance in situational motivation for our predictive models. All participants were informed of the task details before familiarization and provided the option to withdraw without penalty.

### 2.3. Procedures

A familiarization phase preceded the main study. Nine separate two-hour sessions were conducted (9 to 10 participants per session), during which each participant attended one session to practice the long jump and become accustomed to the testing procedures.

The data collection was conducted over a nine-week period from November to January and followed a randomized crossover design. Before the experimental trials, participants completed the electronic version of the SIMS, which was distributed via “Forms.app” to assess their initial motivational state towards engaging in the long jump (their underlying reasons for engaging in the experimental protocol).

Trials commenced in the second week. Following a counterbalancing procedure (see Figure 1 ), participants were divided into two groups: Group 1 (G1) and Group 2 (G2). Over the course of three weeks, a total of 11 long jump testing sessions were conducted (8 to 9 participants per session), each held between 9 a.m. and noon. Throughout the study, the ambient temperature remained remarkably stable, fluctuating only between 10 °C and 14 °C during testing sessions.

During these sessions, participants of G1 completed their long jump trials under normal conditions (NVE), while those of G2 received VE. Following the first three-week experimental phase, a 14-day washout period was implemented to minimize any carryover effects from the experimental conditions. In the final three weeks, the experimental conditions were reversed, with G1 now receiving VE and G2 performing without it. By the end of the experiments, it was necessary to obtain one long jump distance without VE and one with VE from each participant, regardless of exposure order, to ensure their final inclusion in the sample.

VE was delivered by the participants’ peers who were present during the same experimental session, either while they were awaiting their turn or after they had completed their own trials. To standardize the intervention, participants were explicitly instructed to use only three expressions: “Go! Go! Go!”, “Faster! Faster! Faster!”, and “Well Done” (translated from Tunisian Arabic). Participants were directed to address the performing peer by name and deliver the encouragement energetically and vocally (i.e., with a loud voice). The VE was consistently accompanied by rhythmic clapping, with the pace of the clapping instructed to increase in synchronization with the approaching peer’s acceleration during their run-up.

The modal selection of peer-delivered VE over coach-delivered VE was informed by established empirical evidence in similar motor performance contexts [17]. To maximize the ecological validity of the social interaction and promote authentic encouragement, a crucial procedural step was implemented during familiarization sessions: participants were consulted to facilitate their assignment to experimental sessions composed of well-acquainted peers (e.g., existing friendships).

### 2.4. Statistical Analysis

Statistical analyses were conducted using IBM SPSS Statistics (version 29; IBM Corp., Armonk, NY, USA, 2022). Normality was assessed using the Shapiro–Wilk test. Levene’s test was used to verify the equality of variances between groups.

Before conducting the primary comparisons and correlation analyses, a 2 × 2 mixed-design ANOVA was performed to verify the validity of the crossover design and to control for potential order effects. In this model, Condition (With VE vs. Without VE) was defined as the within-subjects factor, and Group (G1: Without VE first vs. G2: With VE first) was defined as the between-subjects factor. This analysis was essential to ensure the 14-day washout period was effective and that no significant carryover, sequence, or period effects biased the results.

Following this validation, paired-samples *t*-tests compared long jump performance without VE and with VE for both sexes separately to determine if the intervention’s effectiveness was consistent across male and female subgroups. Statistical significance was set at *p* < 0.05. Effect size was calculated using Cohen’s *d*.

Relative performance improvement was calculated as:


(1) $$\Delta \%=\frac{Perfromance\;with\;VE-Perfromance\;without\;VE}{Perfromance\;without\;VE}\times 100$$

To examine the predictive power of situational motivation on long jump performance gains (Δ%) and to determine if these relationships were moderated by the participants’ sex, a series of Multiple Linear Regression models were used.

For each of the four subscales of the SIMS (IM, IR, ER, and AM), a separate regression model was constructed using the following equation:


$$\Delta \%=\beta 0+\beta 1\left(SIMS\;subscale\right)+\;\beta 2\left(Sex\right)+\;\beta 3\left(SIMS\;subscale\times Sex\right)+\;\varepsilon$$

In these models, SIMS subscale score (continuous) and Sex (categorical; Male = 1, Female = 2) were entered as primary predictors, alongside their interaction term (SIMS subscale × Sex). The inclusion of the interaction term specifically allowed for the testing of whether the relationship between situational motivation dimensions and performance improvement (Δ%) differed significantly between male and female participants.

The explanatory power of the models was evaluated using the coefficient of determination (R^2^). According to the criteria established by Cohen [21], R^2^ values of 0.02, 0.13, and 0.26 represent small, medium, and large effect sizes, respectively. Given the multifactorial nature of human performance, even small-to-moderate R^2^ values were considered meaningful in this context.

## 3. Results

### 3.1. Validation of the Crossover Design

To verify the validity of the crossover design and account for potential order effects, a 2 × 2 mixed-design ANOVA was conducted with Condition (With VE vs. Without VE) as the within-subjects factor and Group (G1: Without VE first vs. G2: With VE first) as the between-subjects factor.

The analysis revealed a significant main effect of Condition, F_(1, 132)_ = 194.89, *p* < 0.001, η_p_^2^ = 0.596, indicating that long jump performance was significantly higher with VE compared to the control condition.

Importantly, there was no significant main effect for Group, F_(1, 132)_ = 0.74, *p* = 0.391, suggesting no inherent performance differences between the two randomization sequences. Furthermore, the Condition × Group interaction was non-significant, F_(1, 132)_ = 0.59, *p* = 0.443. These results confirm that the 14-day washout period was effective, as the effect of VE did not differ based on the order in which the conditions were administered (i.e., no carryover or period effects were detected).

### 3.2. Effect of VE on Long Jump Performance

As presented in Table 1, mean long jump performance increased under VE for both male and female participants.

Among males (n = 69), performance improved from 4.95 ± 0.55 m (without VE) to 5.08 ± 0.56 m (with VE). A two-tailed paired-samples *t*-test indicated that this increase was statistically significant (*t* = 9.213, *p* < 0.001), with a large effect size (*d* = 1.109).

Similarly, among females (n = 65), performance increased from 3.57 ± 0.54 m to 3.70 ± 0.54 m with VE. This difference was also statistically significant (*t* = 10.731, *p* < 0.001), corresponding to a very large effect size (*d* = 1.331).

Across the full sample, the mean absolute improvement attributable to VE was 0.13 ± 0.11 m, ranging from −0.10 m to +0.47 m. In relative terms, this corresponded to an average increase of 3.24% ± 2.75%, with individual changes ranging from −2.62% to +10.06%. Descriptive statistics for performance changes and situational motivation dimensions are presented in Table 2.

### 3.3. Relationship Between Situational Motivation and the Effect of VE

#### 3.3.1. Intrinsic Motivation (IM) and the Effect of VE

**Figure 3:**
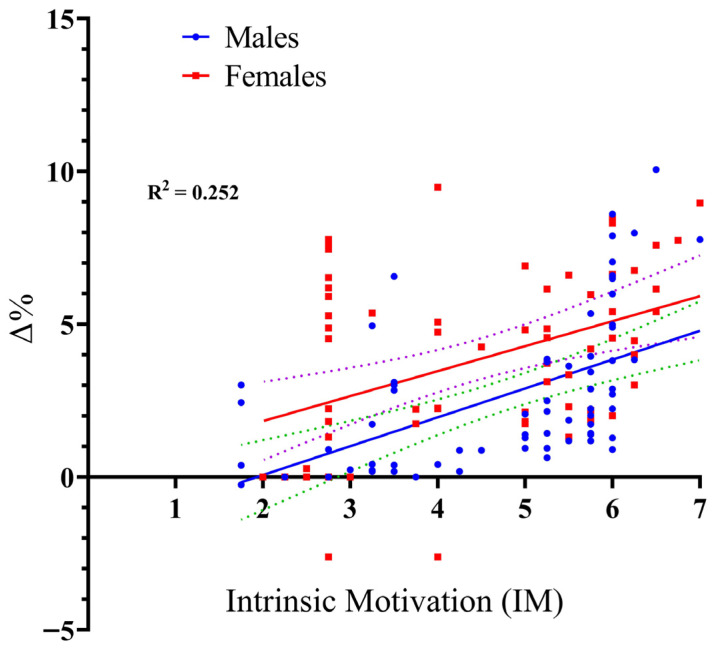
Scatter plot with regression lines illustrating the relationship between Intrinsic Motivation (SIMS score, 1–7) and long jump performance gains (Δ%) for male and female participants. Notes. The solid lines represent the fitted linear regression lines for males and females. The dashed lines represent the 95% confidence intervals around each regression line, indicating the precision of the estimated relationship.

The model for IM was highly significant, F_(3, 130)_ = 14.62, *p* < 0.001, and demonstrated strong explanatory power, accounting for 25.2% of the variance in performance improvement (R^2^ = 0.252). This indicates that IM (β = 0.944, *p* < 0.001) is a primary and reliable driver of the performance gains (Δ%) observed with VE. Regarding the role of the participants’ sex, the analysis confirms that sex did not influence the relationship between IM and performance gains (Δ%). Specifically, the Sex × IM interaction was non-significant (t = −0.434, *p* = 0.665), providing statistical evidence that the positive link between intrinsic motivation and performance gains is universal across sexes (see Figure 3).

#### 3.3.2. Identified Regulation (IR) and the Effect of VE

**Figure 4:**
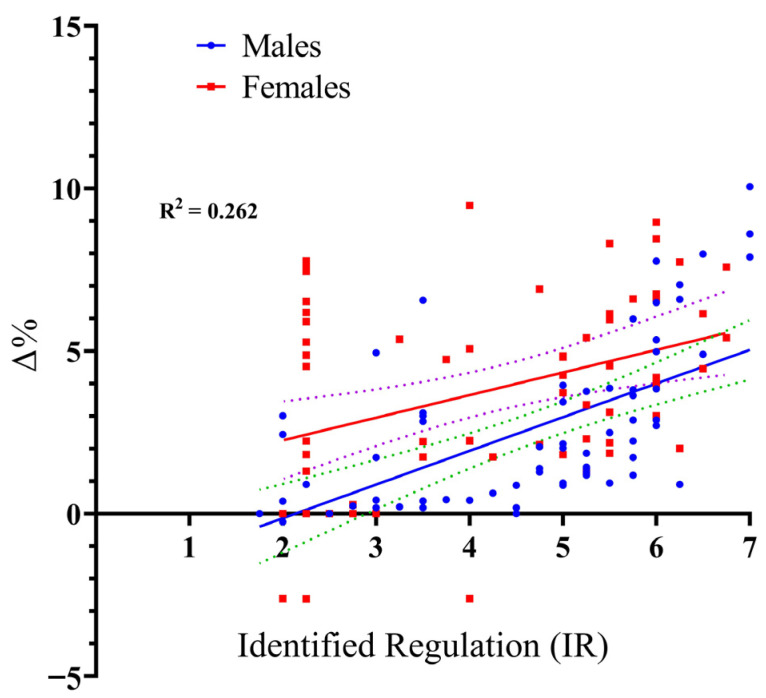
Scatter plot with regression lines illustrating the relationship between Identified Regulation (SIMS score, 1–7) and long jump performance gains (Δ%) for male and female participants. Notes. The solid lines represent the fitted linear regression lines for males and females. The dashed lines represent the 95% confidence intervals around each regression line, indicating the precision of the estimated relationship.

The model for IR was also highly significant, F_(3, 130)_ = 15.37, *p* < 0.001, and demonstrated slightly higher explanatory power, accounting for 26.2% of the variance in performance improvement (R^2^ = 0.262). These results indicate that IR is a robust and reliable predictor (β = 1.036, *p* < 0.001) of the performance gains (Δ%) associated with VE. Consistent with the findings for IM, the participants’ sex did not influence the relationship between IR and performance gains. Specifically, the Sex × IR interaction was non-significant (*t* = −1.213, *p* = 0.227), providing further statistical evidence that the positive link between IR and performance improvement is universal across sexes (see Figure 4).

#### 3.3.3. External Regulation (ER) and the Effect of VE

**Figure 5:**
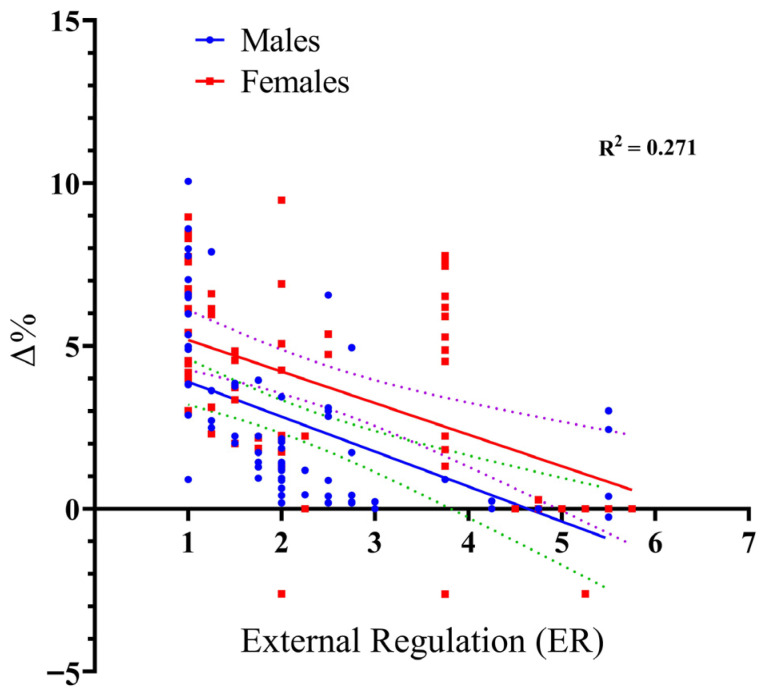
Scatter plot with regression lines illustrating the relationship between External Regulation (SIMS score, 1–7) and long jump performance gains (Δ%) for male and female participants. Notes. The solid lines represent the fitted linear regression lines for males and females. The dashed lines represent the 95% confidence intervals around each regression line, indicating the precision of the estimated relationship.

Following the same systematic approach, we then analyzed the model for ER. The results were highly significant, F_(3, 130)_ = 16.10, *p* < 0.001, and again demonstrated strong explanatory power, accounting for 27.1% of the variance in performance improvement (R^2^ = 0.271). These findings suggest that ER is a reliable predictor of the performance gains (Δ%) associated with VE, though the negative coefficient (β = −1.073, *p* < 0.001) indicates that higher ER scores are associated with lower responsiveness to the VE intervention. Regarding the role of the participants’ sex, the analysis confirms that sex did not influence the relationship between ER and performance gains (Δ%). Specifically, the Sex × ER interaction was non-significant (*t* = 0.32, *p* = 0.749), confirming that the association between ER and performance improvement remains consistent regardless of the participant’s sex (see Figure 5).

#### 3.3.4. Amotivation (AM) and the Effect of VE

**Figure 6:**
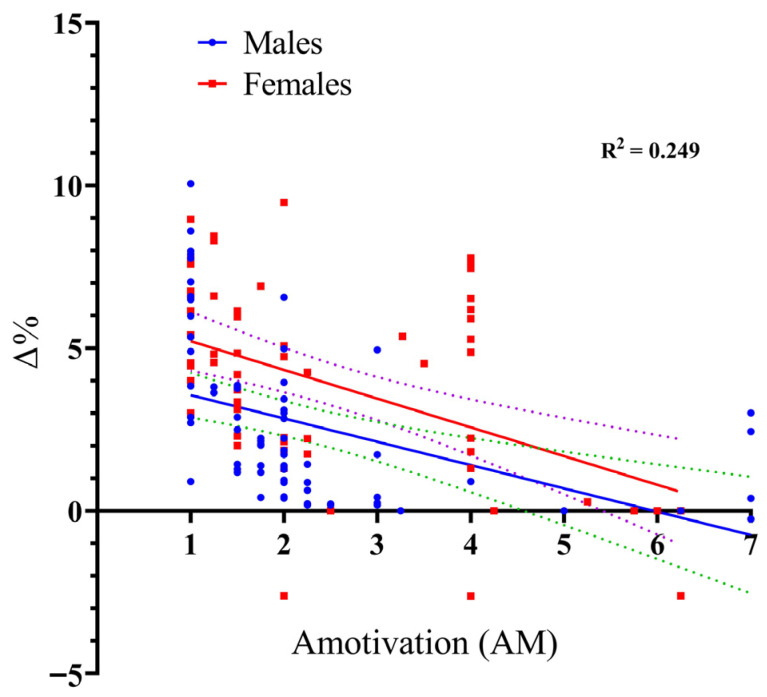
Scatter plot with regression lines illustrating the relationship between Amotivation (SIMS score, 1–7) and long jump performance gains (Δ%) for male and female participants. Notes. The solid lines represent the fitted linear regression lines for males and females. The dashed lines represent the 95% confidence intervals around each regression line, indicating the precision of the estimated relationship.

Finally, the same regression modeling was applied to AM. The model was statistically significant, F_(3, 130)_ = 14.33, *p* < 0.001, accounting for 24.9% of the variance in performance improvement (R^2^ = 0.249). Similar to ER, AM displayed a negative coefficient (β = −0.717, *p* < 0.001), suggesting that AM is associated with reduced responsiveness to the VE intervention. While AM served as a predictor within the overall model, the results confirmed that the participants’ sex did not influence the relationship between this dimension and performance gains (Δ%). Crucially, the Sex × AM interaction was non-significant (*t* = 0.615, *p* = 0.540), providing statistical evidence that the link between AM and performance improvement remains consistent across both male and female participants (see Figure 6).

## 4. Discussion

This study examined whether the performance-enhancing effect of verbal encouragement (VE) in long jump correlates with pre-existing situational motivation in physically active practitioners. The findings support the central hypothesis: Although VE significantly improved performance overall, the magnitude of this improvement was strongly contingent upon the athlete’s initial motivational profile as assessed by the SIMS.

### 4.1. Interpretation of Key Findings

Consistent with a substantial body of empirical research [15,16,18], VE produced a significant enhancement in physical performance. Both male and female participants demonstrated superior long jump distances under peer-delivered VE compared to the non-encouragement condition. These results extend prior findings to explosive track-and-field performance and reinforce the robustness of VE as a performance-enhancing stimulus across task types.

Considering the magnitude of the observed effects is particularly important, especially in light of the reported significant effect sizes (Cohen’s d > 1). The distinctive characteristics of the intervention and the sample may account for the significant effect sizes seen in this study. The long jump task’s explosive character, the highly interactive testing environment, and the application of peer-delivered encouragement among familiar individuals may have all contributed to the performance response. However, these characteristics should be taken carefully because they might have contributed to impact sizes that are bigger than those usually reported in more controlled or less socially engaging contexts.

Several complementary mechanisms may explain this effect. From a psychobiological perspective, VE can function as an external reinforcement stimulus that increases arousal regulation and perceived effort mobilization [22]. According to motivational intensity theory [23], effort investment rises when external cues signal that success is both important and attainable. In parallel, self-efficacy theory [24], posits that supportive verbal input enhances perceived competence, thereby facilitating motor output and persistence. VE may also elevate sympathetic activation and neuromuscular readiness, optimizing explosive force production [25,26]. Furthermore, social facilitation theory [27] suggests that the presence of an encouraging agent enhances dominant responses in well-learned motor tasks. Finally, attentional control frameworks indicate that VE may direct attention toward task-relevant cues while reducing internal interference, thereby improving motor efficiency [28].

The principal contribution of this study, however, lies in demonstrating that VE does not exert uniform effects across individuals. Rather, performance gains were positively correlated with autonomous forms of situational motivation (intrinsic motivation and identified regulation) and negatively correlated with controlled forms (external regulation and amotivation). Participants who engaged in the task out of genuine interest or personal value exhibited the greatest responsiveness to VE. Given that participants were unaware of the specific task during recruitment, expectation-related bias was minimized, strengthening the validity of these associations.

These findings align closely with the SDT [6]. This theory differentiates autonomous motivation, characterized by volition and self-endorsement, from controlled motivation, driven by external contingencies [8]. When VE is delivered to autonomously motivated participants, it is likely interpreted as informational and competence-supportive feedback rather than as controlling pressure. Such interpretation may optimize arousal, attentional focus, and motor coordination, thereby amplifying performance benefits [27,28].

Conversely, participants high in external regulation or amotivation demonstrated attenuated, or occasionally negative performance responses to VE. When engagement is rooted primarily in compliance or a lack of intentionality, external encouragement may fail to enhance effort mobilization and may even be perceived as evaluative pressure. The coherent divergence between autonomous and controlled motivational profiles reinforces the construct validity of the SIMS assessment and supports the interpretation that situational motivation meaningfully correlates with responsiveness to external encouragement. Collectively, these findings position VE not as a universally effective stimulus, but as a context-sensitive intervention whose efficacy depends on the practitioner’s underlying motivational state.

Regarding the motivational profiles of the practitioners, it is important to distinguish between the various forms of non-autonomous regulation. In accordance with the validation of the SIMS by Guay et al. [7], our analysis treated external regulation and amotivation as the non-determined end of the self-determination continuum. However, we acknowledge the theoretical distinction between these two constructs: while external regulation involves performing an activity to satisfy an external demand or reward, amotivation represents a complete lack of intentionality or perceived competence. Our results suggest that both dimensions play a significant role in how a practitioner perceives and utilizes verbal encouragement. By examining these subscales within a multivariable regression framework, we were able to account for their shared variance while respecting their unique psychological contributions to performance gains.

### 4.2. Limitations

Despite the robust findings, this study is subject to limitations that should be considered when interpreting the results and designing future research. First, the study employed a short-duration, explosive, single-attempt task. The interaction between situational motivation and VE may differ in endurance activities, repeated-effort protocols, or team-sport contexts where fatigue accumulation, pacing strategies, and interpersonal dynamics play a larger role. Second, the “dose” of VE was not objectively standardized. While we maintained ecological validity by using peers to deliver the encouragement, the natural variations in their enthusiasm, volume, and timing may have resulted in different intensities across sessions. Future research can consider using more strictly controlled delivery methods, such as pre-recorded audio, to further isolate the effects of the social bond from the physical delivery of the message. Third, situational motivation was assessed at a single time point immediately prior to task execution. While this timing captured the motivational state at engagement, motivation is inherently dynamic and may fluctuate across trials or sessions. Future research employing repeated assessments or ecological momentary methods would provide a more granular understanding of motivational variability. Fourth, the sample consisted of physically active sports science students rather than elite long jump specialists. Highly trained athletes may exhibit more stable motivational structures and performance consistency, potentially altering responsiveness to VE. Finally, VE was standardized to ensure methodological control. However, real-world coaching communication is typically more individualized and nuanced. Future investigations should explore whether personalized or autonomy-supportive variations in VE yield differential effects. Fifth, while the study maintained high ecological validity, several internal factors could not be fully controlled. Although we strictly standardized the testing window (9 a.m. to 12 p.m.) and prohibited external long jump training to minimize diurnal variability and learning effects, some degree of individual daily fluctuations in athletic readiness is inherent in field-based tasks. Sixth, the absence of physiological or perceptual monitoring (such as Heart Rate, Arousal scales, or Rate of Perceived Exertion) limits our ability to identify the precise mechanisms, whether metabolic or psychological, driving the observed performance gains. Future research can incorporate these measures to further clarify how verbal encouragement modulates the objective and subjective experience of maximal physical effort. Finally, as participants were assessed in small groups within testing sessions, potential session-level clustering effects cannot be entirely ruled out. The analyses assumed independence of observations, which may represent a minor source of bias.

### 4.3. Practical Implications

The present findings carry important implications for coaching practice and performance optimization. The primary practical implication of this research is that VE should be viewed not as a universally effective performance enhancer, but as a strategically applied tool within a larger motivational framework. Given that autonomous motivation strongly correlates with superior responsiveness to VE, interventions should first prioritize creating a mastery-oriented environment that fosters genuine interest (intrinsic motivation) and helps practitioners internalize the personal value (identified regulation) of the task. By cultivating self-endorsed commitment, coaches effectively “prime” the athlete to optimally benefit from external encouragement. Practitioners exhibiting high autonomous motivation can use VE liberally and directly to reinforce competence and effort (e.g., “Great effort, you really focused on your speed!”). Those displaying signs of controlled motivation, however, may find simple, generic VE ineffective or even counterproductive if it is perceived as controlling. In these cases, the focus should shift from performance-based encouragement to strategies that support competence while reducing perceived pressure, tailored to the practitioner’s individual needs.

## 5. Conclusions

This study examined the relationship between pre-existing situational motivation in physically active sports science students and their responsiveness to VE during a long jump task. The findings indicate that while VE acts as a robust performance enhancer in the long jump, its efficacy is highly heterogeneous and associated with the motivational state of the participants. Specifically, the findings suggest that VE functions most powerfully as a motivational amplifier when practitioners are driven by autonomous forms of motivation, characterized by intrinsic interest and identified regulation. Conversely, the negative correlation observed between VE effectiveness and controlled motivation (external regulation and amotivation) demonstrates that reliance on external encouragement is significantly limited when an athlete’s engagement is rooted in compliance or a lack of personal volition. Ultimately, these results underscore that VE is not a “magic stick” or a generic solution, but rather a targeted intervention whose impact is correlated with the athlete’s pre-existing psychological architecture.

## Figures and Tables

**Table 1:** Effect of peer verbal encouragement (VE) on long jump performance, separated by sex, including paired *t*-test results.

	Without VE		With VE		Paired-Samples *t*-Test (Two-Tailed)		Effect Size (Cohen’s ***d***)
	M	SD	M	SD	*t*	*p*	
Males (*n* = 69)	4.95 m	0.55 m	5.08 m	0.56 m	9.213	<0.001	1.109
Females (*n* = 65)	3.57 m	0.54 m	3.70 m	0.54 m	10.731	<0.001	1.331

Notes. VE = verbal encouragement; M = mean; SD = standard deviation; t = paired-samples *t*-test statistic; *p* = significance level; d = Cohen’s d effect size.

**Table 2:** Descriptive statistics for the effect of verbal encouragement (VE) on long jump performance and the four dimensions of situational motivation (SIMS subscales).

Variables		M	SD	Min	Max
Effect of VE on long jump performance	Δ	0.13 m	0.11 m	−0.10 m	0.47 m
	Δ%	3.24%	2.75%	−2.62%	10.06%
Dimensions of situational motivation (SIMS subscales)	Intrinsic motivation (IM)	5.11 pts	1.28 pts	1.75 pts	7.00 pts
	Identified regulation (IR)	5.02 pts	1.30 pts	1.25 pts	7.00 pts
	External regulation (ER)	1.90 pts	1.18 pts	1.00 pts	6.00 pts
	Amotivation (AM)	1.99 pts	1.38 pts	1.00 pts	7.00 pts

Notes. VE = verbal encouragement; M = mean; SD = standard deviation; Min = minimum; Max = maximum; Δ = absolute change in performance (m); Δ% = relative change in performance (%); pts = points (SIMS).

## Data Availability

The data presented in this study are available on request from the corresponding author. The data are not publicly available due to privacy and ethical restrictions, as they include individual performance metrics and psychological profiles of student athletes.

## Abbreviations

VE	Verbal encouragement
SIMS	Situational Motivation Scale
IM	Intrinsic motivation
IR	Identified regulation
ER	External regulation
AM	Amotivation
SDT	Self-Determination Theory
M	Mean
SD	Standard deviation
Min	Minimum
Max	Maximum
Δ	Absolute change in performance
Δ%	Relative change in performance
*t*	*t*-test statistic
*p*	Significance level (*p*-value)
*d*	Cohen’s d (effect size)
R^2^	Coefficient of determination
η_p_^2^	Partial eta-squared
